# Inhibition of Oesophageal Squamous Cell Carcinoma Progression by *in vivo *Targeting of Hyaluronan Synthesis

**DOI:** 10.1186/1476-4598-10-30

**Published:** 2011-03-23

**Authors:** Sören Twarock, Till Freudenberger, Eva Poscher, Guang Dai, Katharina Jannasch, Christian Dullin, Frauke Alves, Klaus Prenzel, Wolfram T Knoefel, Nikolas H Stoecklein, Rashmin C Savani, Bernhard Homey, Jens W Fischer

**Affiliations:** 1 Institut für Pharmakologie und Klinische Pharmakologie, Universitätsklinikum Düsseldorf, Heinrich-Heine Universität Düsseldorf, 40225 Düsseldorf, Germany; 2 Abteilung Hämatologie/Onkologie, Zentrum Innere Medizin, Universitätsklinikum Göttingen, 37075 Göttingen, Germany; 3 Klinik für Visceral- und Gefäßchirurgie der Universität zu Köln, 50937 Köln, Germany; 4 Klinik für Allgemein-, Viszeral- und Kinderchirurgie, Universitätsklinikum Düsseldorf, 40225 Düsseldorf, Germany; 5 Divisions of Pulmonary & Vascular Biology and Neonatal-Perinatal Medicine, Department of Pediatrics, The University of Texas Southwestern Medical Center, 75390 Dallas, Texas, USA; 6 Hautklinik, Universitätsklinikum Düsseldorf, 40225 Düsseldorf, Germany

## Abstract

**Background:**

Oesophageal cancer is a highly aggressive tumour entity with at present poor prognosis. Therefore, novel treatment options are urgently needed. Hyaluronan (HA) is a polysaccharide present in the matrix of human oesophageal squamous cell carcinoma (ESCC). Importantly, in vitro ESCC cells critically depend on HA synthesis to maintain the proliferative phenotype. The aim of the present study is (1) to study HA-synthase (HAS) expression and regulation in human ESCC, and (2) to translate the *in vitro *results into a mouse xenograft model of human ESCC to study the effects of systemic versus tumour targeted HAS inhibition on proliferation and distribution of tumour-bound and stromal hyaluronan.

**Methods:**

mRNA expression was investigated in human ESCC biopsies by semiquantitative real-time RT PCR. Furthermore, human ESCC were xenografted into NMRI nu/nu mice. The effects on tumour progression and morphology of 4-methylumbelliferone (4-MU), an inhibitor of HA-synthesis, and of lentiviral knock down of HA-synthase 3 (HAS3), the main HAS isoform in the human ESCC tissues and the human ESCC cell line used in this study, were determined. Tumour progression was monitored by calliper measurements and by flat-panel detector volume computed tomography (fpVCT). HA content, cellular composition and proliferation (Ki67) were determined histologically.

**Results:**

mRNA of HAS isoform 3 (HAS3) was upregulated in human ESCC biopsies and HAS3 mRNA was positively correlated to expression of the epidermal growth factor (EGF) receptor. EGF was also proven to be a strong inductor of HAS3 mRNA expression *in vitro*. During the course of seven weeks, 4-MU inhibited progression of xenograft tumours. Interestingly, remodelling of the tumour into a more differentiated phenotype and inhibition of cell proliferation were observed. Lentiviral knockdown of HAS3 in human ESCC cells prior to xenografting mimicked all effects of 4-MU treatment suggesting that hyaluronan produced by ESCC is accountable for major changes in tumour environment *in vivo*.

**Conclusions:**

Systemic inhibition of HA-synthesis and knockdown of tumour cell HAS3 cause decreased ESCC progression accompanied by tumour stroma remodelling and may therefore be used in novel approaches to ESCC therapy.

## Background

Oesophageal cancer is the sixth leading cause of cancer deaths worldwide [1]. The mortality rate associated with oesophageal cancer is similar to its incidence rate because of its generally advanced stage at the time of diagnosis, its aggressive characteristics, and because of the paucity of effective treatment strategies. In spite of its poor prognosis, oesophageal cancer has not been well studied [2]. Two types of oesophageal cancer exist: adenocarcinoma, and oesophageal squamous cell carcinoma (ESCC), which corresponds to approximately 50% of all oesophageal cancers. Standard treatment for oesophageal cancer comprises surgery, chemoradiotherapy, and palliative chemotherapy with cisplatin, fluorouracil, and taxanes. However, the response to chemotherapy typically lasts only a few months, and the median survival time is less than one year [3]. Recent technical advances in surgery, the use of neoadjuvant chemoradiotherapy, and new cytotoxic drugs have increased the response rates but have had no meaningful effect on survival.

Hyaluronan (HA) is an unbranched high molecular weight polysaccharide that is composed of D-glucuronic acid beta(1-3)-D-N-acetyl-glucosamine beta(1-4). HA is produced by three isoforms of the hyaluronan synthase family (HAS1-3), which are located at the plasma membrane and extrude the growing HA polymer into the extracellular space [4]. Overexpression of either HAS2 or HAS3 in several tumour types such as prostate cancer [5], breast cancer [6,7], osteosarcoma [8] and colon carcinoma [9] is known to be associated with higher malignancy or metastasis. The activity of all three HAS isoenzymes can be inhibited by 4-methylumbelliferone (4-MU), which depletes the activated uridine diphosphate-glucuronic acid precursor pool and thus leads to decreased HA production [10]. Recently, 4-MU has been studied in different animal models and was shown to inhibit liver metastases of melanoma cells [11], to enhance chemotherapeutic action in pancreatic and breast cancer cells [12,13] and to attenuate tumour progression along with induction of apoptosis in prostate cancer cells [14].

HA activates membrane receptors such as the receptor of HA-mediated motility (RHAMM) and CD44 to induce signalling and specific cellular responses. Both CD44 and RHAMM have been implicated in tumour cell biology and tumour progression [15].

An HA-rich matrix is important for a variety of aspects of tumour pathobiology including anchorage-independent growth, migration, angiogenesis, suppression of apoptosis [15,16] and metastasis [8,17]. Recently strong evidence for the importance of HA in the microenvironment of tumours and in the tumour stroma has been presented [18,19]. A variety of different types of cancer are characterised by either high amounts of tumour cell- associated HA (e.g., colon and gastric cancer) or high amounts of stromal HA (e.g., breast, ovarian, and prostate cancer) or both. In some of these malignancies (e.g., colon cancer), tumour-associated HA is an independent prognostic factor for poor outcome [20]. In other tumours (e.g., ovarian and prostate cancer) it is the stromal HA that is correlated with poor outcome, most likely because of the accelerated growth of the tumours and their metastases [4,21]. With respect to oesophageal cancer it is has been demonstrated that HA accumulates in the parenchyma and stroma [22].

The HA matrix of oesophageal carcinoma may contain novel targets for therapeutic approaches such as the HAS-isoforms, hyaluronidases and HA-receptors. Furthermore, the role of individual HAS enzymes and the factors that regulate HAS expression in oesophageal cancer have not been defined. In addition, the relative importance of stromal versus tumour cell HAS expression has not been addressed experimentally in any cancer yet, which is due to the fact that HAS2 deficient mice are lethal and HAS1 and HAS3 deficient mice are not available to the scientific community [23].

Previously it was demonstrated in ESCC cell lines that HA-synthesis mediated by HAS3, and to a lesser extent by HAS2, is required for the malignant cell phenotype characterised by filopodial plasma membrane extensions and high proliferative activity [24]. Knockdown of HAS3 and inhibition of HA-synthesis by the small molecule inhibitor, 4-MU, caused a rapid loss of focal contacts which was followed by resolution of filopodia and inhibition of proliferation and migration. Therefore, the aim of the present study was to elucidate whether HAS isoforms are specifically upregulated in human ESCC tumour specimens and if so whether inhibition of HA synthesis would be effective to inhibit tumour growth *in vivo*. Furthermore changes in tumour morphology and distribution of HA and HA receptors, following either systemic HA inhibition by 4-MU or inhibition of tumour HA production by lentiviral knockdown of HAS3, were examined. This approach may help to define and specify the molecular targets and to explore the therapeutic promises of pharmacologic HAS inhibition in ESCC.

## Methods

### Reagents and substances

Unless otherwise stated, all reagents were obtained from Sigma-Aldrich, Munich, Germany. Erlotinib was bought from LC Laboratories, Woburn. MA, USA. Cetuximab is a product of Merck Serono, Darmstadt, Germany.

### Cell culture

OSC1 cells were a gift from M. Sarbia [25] and were used for xenograft and cell culture experiments throughout the present study. The human foreskin fibroblast cell line Hs68 used in the co-culture experiments was purchased from ATCC (Wesel, Germany). OSC1 and Hs68 cells were maintained as monolayer cultures in RPMI-1640 (Sigma-Aldrich) supplemented with 10% fetal bovine serum, L-glutamine, penicillin, and streptomycin at 37°C, 5% CO_2 _and 95% humidified air.

### Human ESCC specimens

Tissue samples from oesophageal squamous cell carcinomas (ESSC, n = 20) and normal oesophageal mucosa (n = 13) were collected from patients undergoing radical en bloc oesophagectomy at Düsseldorf University Hospital. The tissues were snap-frozen in liquid nitrogen immediately after resection and stored in liquid nitrogen until use. Written informed consent was obtained from all patients. The collection of the fresh tumour samples was approved by the ethics committee of the Heinrich Heine University Düsseldorf. Tumour stage and grading were classified by routine histopathologic assessment according to the UICC (Union Internationale Contre le Cancer) Classification for Malignant Tumours; the pathologists performing the assessment were unaware of the experimental data.

### Xenograft Model

NMRI nu/nu mice were used for subcutaneous tumour formation experiments after xenografting of OSC1 cells with or without previous lentiviral transduction *in vitro*. The tumours were initiated by the subcutaneous injection of 10^6 ^OSC1 cells into both flanks, and the mice were monitored after xenografting for 47 days in the 4-MU group and 65 days in the shHAS3 group, respectively. The mice were treated with 4-MU, which was pelleted into the chow, at a daily dose of 250 mg per mouse. Treatment started two days before xenografting. Biopsies for immunostaining and molecular studies were taken after sacrifice of the animals at the end of the experiment. The animal experiments were approved by the local animal facility and the *Landesamt für Natur, Umwelt und Verbraucherschutz, NRW*.

### Flat-Panel Detector Volume Computed Tomography Imaging (fpVCT)

After an iodine-containing contrast agent (Isovist 300^®^) was injected intravenously, mice were subjected to fpVCT, a nonclinical volume CT prototype (GE Global Research, Niskayuna, NY), as described previously [26].

### Immunostaining

Cryosections (8 μm) were derived from tumour tissue and fixed in acetone-methanol (2:3) for the following immunostaining: human cytokeratin 18 (Progen Biotechnik, Heidelberg, Germany; 1:200), alpha-smooth muscle actin (Abcam; 1:50), secondary antibodies, goat anti-guinea-pig-FITC, goat anti-rat-RhodX (Dianova, Hamburg, Germany; 1:200), and sheep anti-rabbit-Cy3 (Sigma-Aldrich; 1:200); biotinylated HABP was detected with Cy3-labeled streptavidin (Dako; 1:200). Alternatively, fixation with 96% ethanol was used for staining with rabbit anti-human Ki67 (Abcam; 1:50) and rabbit anti- CD44 (Sigma-Aldrich; 1:1000), which were detected with sheep anti-rabbit-IgG F(ab)2-Cy3 (Sigma-Aldrich; 1:50/1:500). HA was detected by biotinylated HABP (Seikagaku, Tokyo, Japan; 6 μg/ml) and FITC- or Cy3-labeled streptavidin (Dako Deutschland, Hamburg, Germany;1:200). Nuclei were counterstained by Hoechst 33342 (Invitrogen, Karlsruhe, Germany; 1:20000). Cell culture experiments were carried out on glass cover slides, fixed with 3.7% formalin solution and stained as above. Ki67 and HA staining were quantified by using ImageJ 1.37c software with the nucleus counter plug-in. For quantification five randomly selected images from each tumour section were analyzed and the average was used as *n *equals one.

### Real-time RT-PCR

The PCR reactions were performed according to standard procedures with the SYBR Green PCR Master Mix (Applied Biosystems). Relative expression levels were compared by using real-time PCR with the 2^[-ΔΔC(T)] ^method. The primer sequences of the genes of interest are given in Table 1[27,28].

**Table 1:** Primer sequences used for quantification of gene expression

Gene	Primer sequence
human EGFR	5'-GGA GAA CTG CCA GAA ACT GAC C-3'
	5'-GCC TGC AGC ACA CTG GTT G-3'
human GAPDH	5'-GTGAAGGTCGGAGTCAACG-3'
	5'-TGAGGTCAATGAAGGGGTC-3'
human HAS2	5'-GTGGATTATGTACAGGTTTGTGA-3'
	5'-TCCAACCATGGGATCTTCTT-3'
human HAS3	5'-GAGATGTCCAGATCCTCAACAA-3'
	5'-CCCACTAATACACTGCACAC-3'

### Lentiviral knockdown of HAS3

The ESCC cell line, OSC1, was used for xenografting and was maintained as described [25] in monolayer cultures. HAS3 knockdown was achieved by using the MISSION™ Lentiviral shRNA knockdown system (Sigma-Aldrich). The used hairpin sequence was 5'-CCGGGCTCTACAACTCTCTGTGGTTCTCGAGAACCACAGAGAGTTGTAGAGCTTT TTG-'3. A scrambled shRNA was used as a control. The transfer into the packaging line HEK 293T (ATCC, LGC Standards, Wesel, Germany) was performed with the lipofection reagent Fugene 6 (Roche, Grenzach-Wyhlen, Germany). After 16 h, the medium was changed to Iscove's Modified Dulbecco's Medium (IMDM) for better stability of the produced lentiviral particles. The next day, the lentiviruses were harvested and concentrated by centrifugation with poly-l-lysine under the conditions reported previously [29]. After verification of HAS3 mRNA knockdown by RT-PCR target cells were transfected at a multiplicity of infection (MOI) of 10 and kept for 5 days in normal growth medium before injection.

### Statistical Analysis

Statistical analysis of mRNA levels in biopsy samples was performed by using the nonparametric Mann-Whitney test and the Spearman correlation analysis. All other datasets were analyzed either by ANOVA and the Bonferroni post hoc test or by Student's *t *test as appropriate. Data are presented as means ± SEM. Statistical significance was assigned at the level of p < 0.05.

## Results

### HAS3 is upregulated in human oesophageal SCC biopsies and correlates with EGF receptor expression

**Figure 1:**
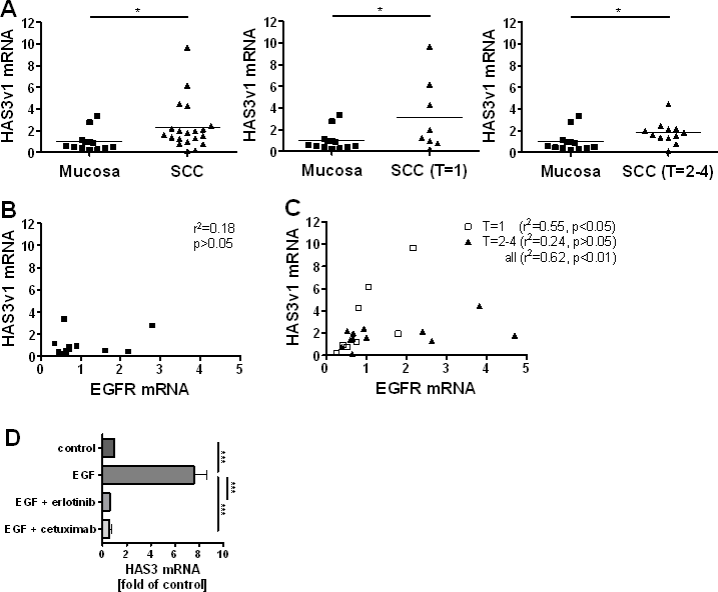
**HAS3 was upregulated and correlated with EGFR in human ESCC**. (**A**) Real-time PCR of HAS3 mRNA from biopsy specimens from normal mucosa (*n *= 13) and human ESCC differentiated into TNM staging (defining tumour size (T), lymph node involvement (N) and existence of metastases (M)) (**n **= 20), mean ± SEM, *, p < 0.05. (**B**, **C**) EGFR expression was determined in the same biopsy specimens as in (A) and correlated to HAS3 expression in normal mucosa (**B**) and in ESCC (**C**). HAS3 expression was significantly correlated to EGFR expression in ESCC. (**D**) EGF stimulated HAS3 mRNA expression in the human ESCC cell line OSC1. This effect was rescued by use of the EGFR tyrosine kinase inhibitor erlotinib and the molecular anti-EGFR antibody cetuximab. Data are mean ± SEM; n = 3-5; ***, p < 0.001.

We analysed the expression of HAS1-3 in human ESCC tumours by RT-PCR and compared to healthy oesophageal mucosa. HAS3 was the main isoform of the studied ESCC tumour samples. This result is in accordance with the HAS expression pattern found in the ESCC cell line OSC1 as determined earlier [28]. Therefore, OSC1 cells were used in this study for *in vitro *experiments and for the xenograft model. In addition, only HAS3 expression was significantly higher in ESCC than in normal mucosal tissue whereas there was no significant increase regarding HAS1 and HAS2 (data not shown). This result was true over all studied samples (2.34+-0.5 fold induction vs. control, mean+-SEM, p < 0.05) as well as for the T = 1 (3.15+-1.2 fold induction vs. control, mean+-SEM, p < 0.05) and the T = 2-4 (1.81+-0.3 fold induction vs. control, mean+-SEM, p < 0.05) subgroups according to TNM classification (defining tumour size (T), lymph node involvement (N) and existence of metastases (M)) (Figure 1A). Furthermore, the mRNA levels of HAS3 were positively correlated with the mRNA levels of EGF receptor (HER1, ErbB1) in tumour cells, but no correlation between these mRNA levels was observed in normal mucosa (Figure 1B, C). Interestingly, T1 grade tumour samples showed a steeper correlation than did T2-4. This might indicate a stronger dependence of early tumour grades on EGF pathway signalling to maintain HAS3 activity. In line with these findings, EGF receptor activation led to induction of HAS3 in ESCC cells, which could be rescued by use of the EGF receptor tyrosine kinase inhibitor erlotinib and the monoclonal anti-EGFR antibody cetuximab (Figure 1D).

### 4-MU inhibits tumour growth *in vivo *and causes tumour-stroma remodelling

**Figure 2:**
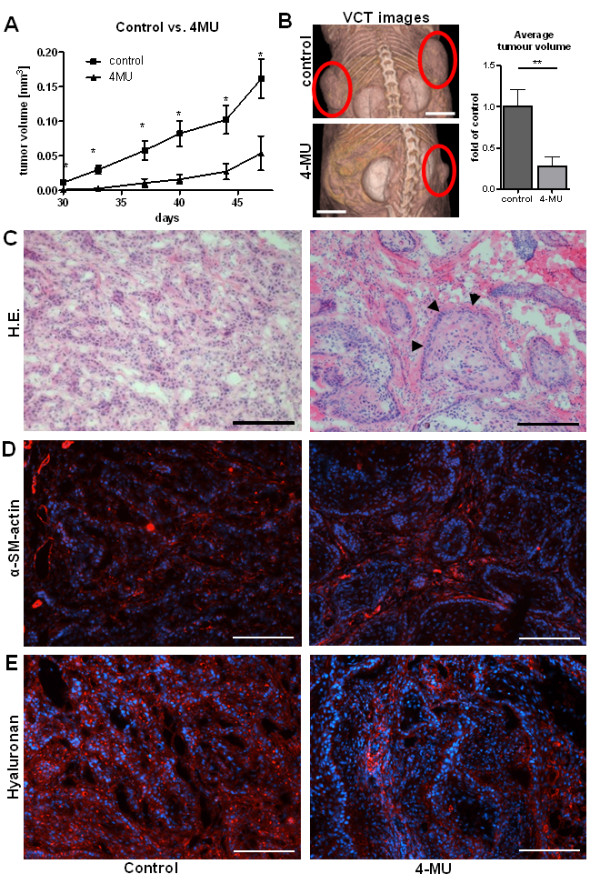
**4-MU treatment inhibited tumour growth of OSC1 cells in nude mice**. Nude mice received subcutaneous injection of 10^6 ^OSC1 cells, and tumour growth was monitored for 47 consecutive days. (**A**) Tumour volume as determined by calliper measurements; mean ± SEM, *n *= 21 (control), 17 (4MU); *, p < 0.05. (**B**) Representative fpVCT images of control mice and 4-MU treated mice. Scale bars, 1 cm. Average tumour volumes after sacrification of mice at the end of the experiment as measured by fpVCT showed a significant reduction in tumour volume in the 4-MU group. (**C**) Cryosections stained with H&E demonstrated a differentiated tumour phenotype in mice treated with 4-MU. This phenotype was characterised by larger tumour cell clusters with a cell-rich border region (arrows) and pronounced tumour stroma. Scale bars, 200 μm. (**D**) Stromal fibroblasts were stained with alpha-smooth muscle actin staining (red). Scale bars, 200 μm. (**E**) Hyaluronan staining (red) was associated with both tumour cells and stromal tissue and was markedly reduced by treatment with 4-MU. Scale bars, 200 μm. Shown are representative images of n = 6 (control) and n = 7 (shHAS3) experiments.

A xenograft tumour model was established by subcutaneously injecting the human ESCC line OSC1 into the flanks of NMRI nu/nu mice. The mice were given oral doses of the small molecule HAS inhibitor 4-MU starting 2 days before injection for the whole experimental period. During the first 47 days after xenografting calliper measurements showed that treatment with 4-MU strongly inhibited the time course of tumour progression (Figure 2A). At the end of the experimental period additional analysis using flat-panel volume computed tomography (fpVCT) revealed also significantly lower tumour volumes (control 100 ± 20,0% vs. 4-MU 27,4 ± 11,8%, Figure 2B).

Treatment with 4-MU not only was associated with decreased tumour size but also caused remarkable alterations in tumour morphology. Histopathological examination of tumour specimens from control mice showed that OSC1-derived xenograft tumours were poorly differentiated, with numerous loosely cohesive tumour cells (Figure 2C, left). In contrast, tumours from mice treated with 4-MU were characterised by the formation of distinct tumour cell clusters and large continuous areas of intratumoural stroma, as indicated by alpha-smooth muscle actin staining (Figure 2D, right). The outer circumference of the clusters exhibited a cell-rich border region (Figure 2C, right, arrows). Staining with the HABP probe showed that HA was found in the tumours but at levels lower in mice treated with 4-MU than in control mice (Figure 2E).

### Knockdown of HAS3 expression in OSC1 cells is sufficient to inhibit tumour progression and to mimic the morphological stroma redistribution as caused by systemic HAS inhibition

**Figure 3:**
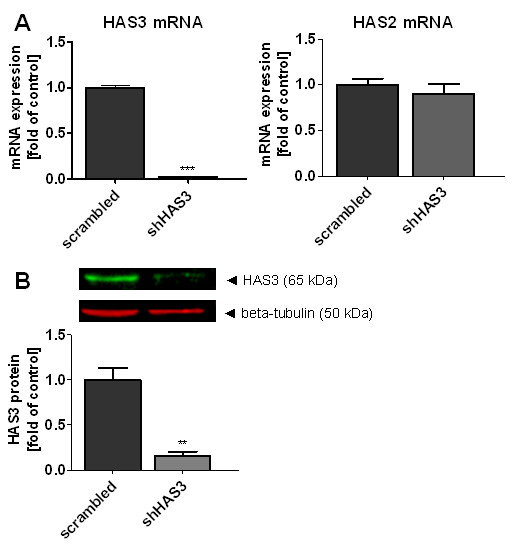
**Lentiviral knockdown of HAS3 in OSC1 cells**. The transduction with lentiviral shHAS3 results in (**A**) a strong decrease of HAS3 mRNA without affecting HAS2 mRNA levels. (**B**) A significant reduction of HAS3 protein levels was found. Shown are the results of three independent RT-PCR experiments and a representative immunoblot of HAS3 with quantitative analysis of n = 3 experiments, mean ± SEM, ** p < 0.01, *** p < 0.001.

HAS3 is the major isoform in human ESCC as determined by real time RT-PCR and was correlated to EGFR expression, perhaps pointing to the functional importance of HAS3 in ESCC. Because the systemic application of 4-MU inhibits HA synthesis in both tumour cells and stromal fibroblasts independently of the involved HAS isoforms, the relative contribution and functional significance of HA derived specifically from tumour cell associated HAS3 was addressed. Transduction with shHAS3 lentivirus caused marked knockdown of HAS3 mRNA and protein expression (Figure 3).

**Figure 4:**
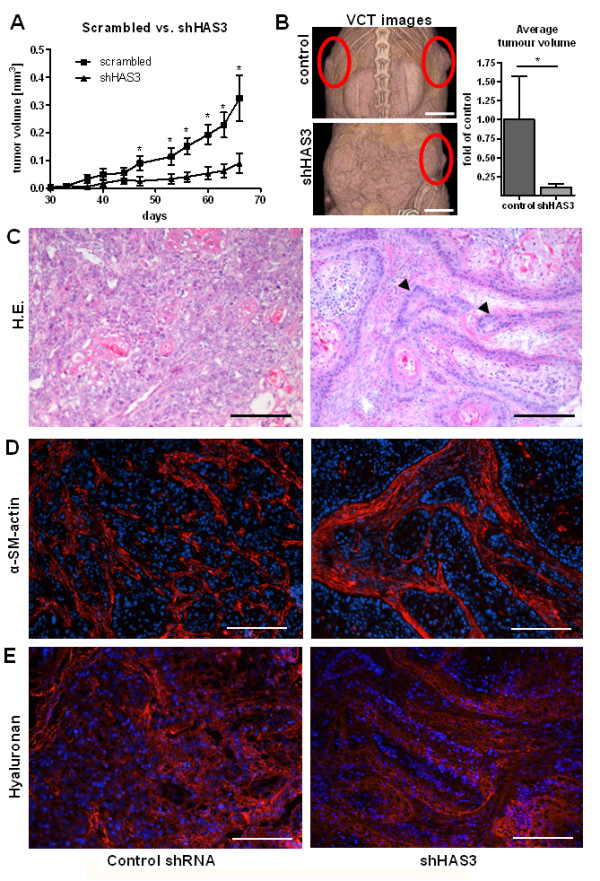
**Lentiviral knockdown of HAS3 in OSC1 cells inhibited tumour growth after xenografting of transduced cells into nude mice**. (**A**) Nude mice received subcutaneous injections of 10^6 ^OSC1 cells that had been transduced *in vitro *with a lentivirus containing either scrambled shRNA (control) or shHAS3. Tumour growth as assessed by calliper measurements; mean ± SEM, *n *= 6 (control), 7 (shHAS3); *, p < 0.05. (**B**) Representative fpVCT images of mice after 65 days. Average tumour volumes after sacrification of mice at the end of the experiment as measured by fpVCT showed also a significant reduction in tumour volume in the shHAS3 group. (**C**) H&E revealed changes in tumour morphology strikingly similar to those after 4-MU (compare Figure 2). (**D**) Tumour stroma was visualised by alpha-smooth muscle actin staining (red). (**E**) Xenografting shHAS3 tumour cells caused redistributed hyaluronan staining specifically localised to the outer layer of tumour cell clusters. Scale bars, 200 μm. Shown are representative images of n = 6 (control) and n = 7 (shHAS3) experiments.

The subcutaneous injection of the shHAS3 transduced OSC1 cells into nu/nu mice resulted in a marked inhibition of tumour growth (control 100 ± 57.7% vs. shHAS3 10.1 ± 5.3%, Figure 4A, B) and in a tumour morphology strikingly similar to that seen after systemic inhibition of HA synthesis. Specifically, tumours derived from shHAS3-transduced OSC1 cells exhibited a phenotype characterised by large tumour cell clusters with condensed cell-rich borders (Figure 4C, arrows) whereas the morphology of control tumours was characterised by numerous small clusters of OSC1 cells (Figure 4C, left).

**Figure 5:**
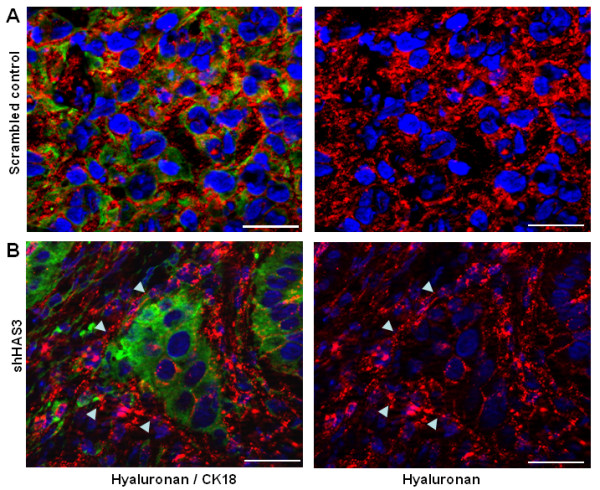
**Detection of the distribution of HA in shHAS3 knockdown xenografts by double staining for tumour cells (CK18) and HA**. Section from the same series as in Figure 3 were double stained for HA (red) and CK18 (green) to determine the exact location of HA in the xenograft tumour sections. (**A**) Scrambled control sections revealed a strong HA signal in tumour and stroma. (**B**) HA staining in shHAS3 samples showed a strong signal in the stroma and tumour/stroma interface (arrows). However, HA was much less pronounced within the tumour cell clusters. Scale bars, 30 μm. Shown are representative images of n = 6 (control) and n = 7 (shHAS3) experiments.

Furthermore, alpha-smooth muscle actin staining showed that stromal tissue was strongly pronounced in shHAS3 tumours and separated the large OSC1 cell clusters (Figure 4D). The lentiviral knockdown of HAS3 in the xenografted OSC1 cells resulted in reduced stromal HA staining and in addition in pronounced association of the residual HA with the circumference of tumour cell clusters (Figure 4E). To identify the tumour cells anti-human cytokeratin 18 (CK18) immunostaining was performed in combination with HA staining (Figure 5). Strong stromal HA signals were detected in the vicinity of CK18 positive tumour cell islands in shHAS3 xenografts (Figure 5B, arrows). However, within the tumour cell clusters HA was less pronounced.

In combination, these findings indicate that 4-MU and shHAS3 reduce the growth of OSC1-derived tumours in nude mice, cause a transition to a more differentiated tumour phenotype and cause formation of large tumour cell clusters that were separated by pronounced stromal tissue with reduced HA content.

### Possible role of tumour cell CD44 for maintenance of pericellular HA matrix in OSC1

**Figure 6:**
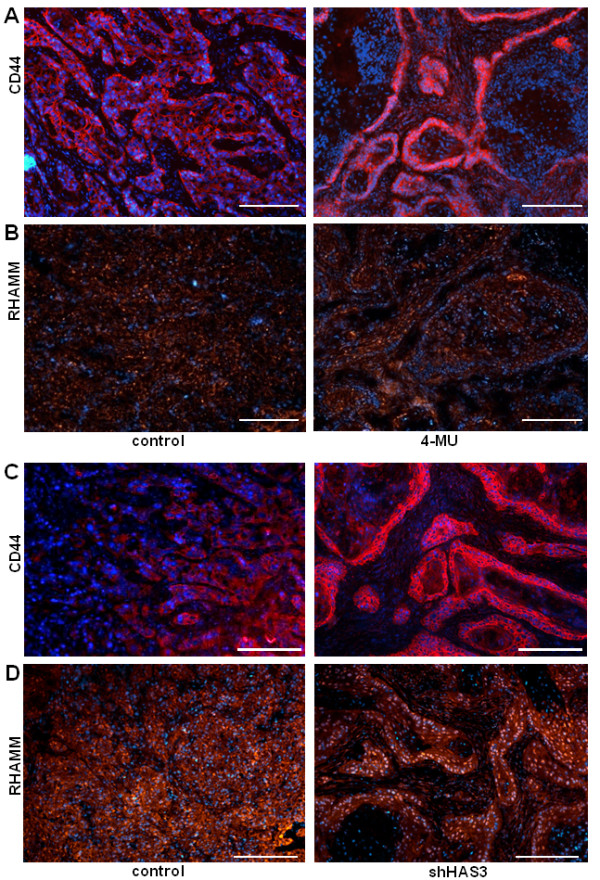
**Expression of CD44 and RHAMM in response to 4-MU versus shHAS3**. (**A**) 4-MU, immunostaining of human CD44 (red) revealed that CD44 was redistributed to the interface between tumour cells and stroma in response to 4-MU. (**B**) 4-MU, RHAMM was detected in both OSC1 cells and stromal cells. (**C**) shHAS3, shHAS3 transduction of tumour cells caused a similar redistribution of human CD44 (red) to the tumour-stroma interface. (**D**) shHAS3, RHAMM (red) was detected in both shHAS3 transduced OSC1 and stromal cells. Scale bars, 200 μm. Shown are representative images of n = 6 (control) and n = 7 (shHAS3) experiments.

**Figure 7:**
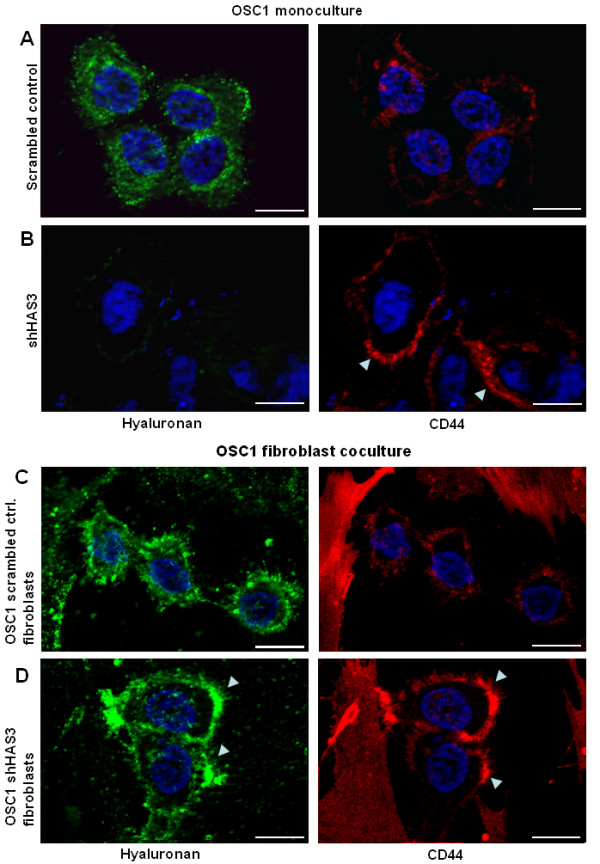
**Co-culture of OSC1 cells and fibroblasts suggested binding of stromal HA to the tumour cell surface via CD44**. OSC1 cells were cultured alone and with fibroblasts to gain mechanistic insight into the origin of OSC1-bound HA in shHAS3 transduced OSC1 cells. (**A**) In monoculture OSC1 cells showed a strong staining for HA (green) and a weak signal for CD44 (red). (**B**) Transduction with shHAS3 strongly decreased HA deposition (green) and increased CD44 expression (red, arrows). (**C**) In co-cultures OSC1 cells exhibited similar pericellular HA as compared to controls in A. (**D**) In contrast shHAS3 transduced OSC1 cell still exhibited a strong pericellular HA signal (green, arrows) in the presence of fibroblasts that co-localized with increased CD44 (red, arrows). Scale bars, 10 μm. Shown are representative images of n = 3.

Next, immunostaining was used to determine the expression of the HA receptors CD44 and RHAMM in response to treatment with 4-MU and shHAS3. The expression of human CD44 was pronounced in all tumour cells in controls and appeared to be redistributed and upregulated after 4-MU treatment in the tumour cells that faced the stromal tissue (Figure 6A, right). Similar changes in CD44 expression occurred in the shHAS3 group compared to mice that received OSC1 cells transduced with a control vector (Figure 6C, right). RHAMM was strongly expressed in tumour cells and to a weaker extent in stromal cells and did not respond to 4-MU or shHAS3 (Figure 6B, D). Next we considered that upregulated CD44 may bind stromal HA to the tumour cell surface. To further examine this possibility we compared CD44 and HA staining in monoculture of OSC1 with OSC1 and fibroblast co-culture. In monocultures the lentiviral knockdown of HAS3 resulted in an increased CD44 staining similar to the *in vivo *results whereas the pericellular HA signal was hardly detectable (Figure 7A, B). In contrast, in co-cultures of fibroblasts and OSC1 cells, strong pericellular HA signals were obtained in controls (Figure 7C) and were not diminished by knock down of HAS3 in OSC1 (Figure 7D, arrows). These observations suggest that HAS3 depleted OSC1 cells might utilise HA produced by stromal cells by means of increased CD44 expression to maintain the pericellular HA matrix.

### Inhibition of proliferation

**Figure 8:**
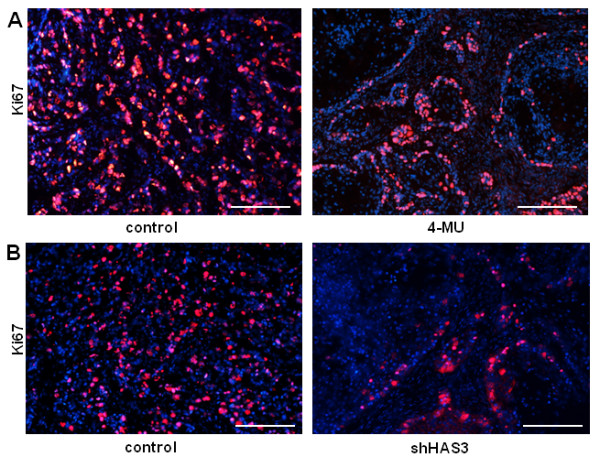
**Effects of 4-MU and tumour cell targeted shHAS3 on proliferation in OSC1 xenografts**. (**A**) 4-MU, staining of human Ki67 (red) was markedly reduced by 4-MU treatment and confined to the interface between tumour cells and stroma. (**B**) shHAS3, staining of human Ki67 (red) was markedly reduced in tumour cells transduced with shHAS3. Scale bars 200 μm. Shown are representative images of n = 6 (control) and n = 7 (shHAS3) experiments.

To address the underlying mechanisms for inhibition of tumour progression, proliferation was determined by immunostaining in the xenograft tumours. Immunostaining of the proliferation marker Ki67 revealed numerous small clusters of proliferating tumour cells in the controls. The proliferative activity was lower in specimens treated with 4-MU than in controls and the proliferating cells were confined to the outer circumference of the large tumour cell clusters that tested positive for HA, CD44 and RHAMM (Figure 8A; proliferating cells in controls, 25% ± 3%; proliferating cells in sections of mice treated with 4-MU, 15% ± 3%; p < 0.05; n = 4-5).

Subsequently the above described staining patterns were compared to mice xenografted with shHAS3 transduced OSC1 cells. The percentage of proliferating tumour cells was lower in shHAS3-transduced tumours compared to control tumours (Figure 8B: control, 35% ± 6%; shHAS3, 20% ± 2%; p < 0.05, n = 7). As observed after 4-MU treatment, the remaining proliferative activity was confined to the CD44-positive circumference of tumour cell islands. These results strongly support the conclusion that inhibition of HAS3-mediated HA synthesis by OSC1, rather than HA synthesis by stromal cells, is sufficient to inhibit ESCC proliferation and progression and to cause stromal remodelling into a more differentiated tumour phenotype. In combination, tumour cell specific knock down of HAS3 pheno-copied the effect of systemic inhibition of HA synthesis.

## Discussion

HA synthesis is not sufficient for malignant transformation [30], but HA-binding proteins and HA receptors provide a matrix environment that supports the malignant phenotype of cancer cells, stromal cell recruitment, and, thus, the progression of cancer [31]. Recently, the importance of stromal HA-binding proteins was demonstrated for the proteoglycan versican, which triggers the invasion and retention of inflammatory cells in Lewis lung carcinoma and supports metastasis [17]. In human ESCC, HA accumulates in the parenchyma and stroma, and HA is produced by both tumour cells and stroma [22,28]. The amount of HA, which is supposed to be initially high in ESCC, decreases with progression to undifferentiated aggressive carcinomas; this finding suggests increased turnover [32].

Amount of HA and distribution are important prognostic factors in a variety of tumour types. However, important differences exist between tumours that originate from different types of tissue. Tumours arising from simple epithelia such as lung [33], gastric [34], salivary gland [35] and from the thyroid epithelium [36] show a strong correlation between tumour stage and increased HA content. In contrast, those derived from stratified epithelia i.e. oral, laryngeal, oesophageal and skin epithelium are characterized by an increase in HA abundance in early tumour stages which decreases in high grade poorly differentiated tumour stages [22,32]. In line with this, a tendency to increased HAS3 levels in the T = 1 stage compared to T = 2-4 stages was also seen in the present work (Figure 1A) although this was not significant.

The experiments reported here were performed to further increase our understanding about the role of HA synthesis in the progression of human ESCC, to evaluate the therapeutic potential of pharmacologic inhibition of HA synthesis for this tumour type and to attempt to differentiate the roles of tumour cell derived HA versus stromal cell-derived HA. Therefore, we analysed the response of ESCC xenografts to systemic versus tumour cell-targeted interference with HA synthesis. The inhibition of ESCC xenograft tumours by 4-MU is in line with reports showing that 4-MU has anti-tumour activity: it inhibits liver metastasis of melanoma cells; sensitises pancreatic cancer cells to gemcitabine and breast cancer cells to trastuzumab treatment in mice; and decreases prostate cancer cell growth in a xenograft model [11-14]. However, this is the first demonstration that inhibition of a specific HAS isoform, HAS3, in tumour cells is as efficient as systemic HAS-inhibition by 4-MU.

Specifically, a more differentiated tumour phenotype, pronounced stromal strands, fewer singular tumour cells and reduced proliferation were observed. This *in vivo *phenotype shows strong similarities to the phenotype observed *in vitro *after treatment with 4-MU or shHAS3: specifically *in vitro *formation of tumour cell clusters with smooth cell borders occurred in response to inhibition of HA synthesis [24]. After knock down of HAS3 xenografted OSC1 cells still exhibited strong pericellular HA staining concomitant with pronounced CD44 staining suggesting that the elevated CD44 expression may cause binding of stroma derived HA to the tumour cell surface. The recruitment of stromal HA in response to knock down of HAS3 by tumour cells might be part of a compensatory mechanism. This thesis is corroborated by reports that melanoma cells stimulate stromal HA production by soluble factors to facilitate tumour growth and invasion [37] and lung carcinoma cells using stimulatory membrane-bound glycoproteins to support locomotion and adhesion [38]. Functionally, the HA/CD44 interactions might contribute to tumour cell proliferation, because after inhibition of HA synthesis by 4-MU or application of shRNA targeting HAS3 the remaining proliferative activity of tumour cells was confined to the CD44 positive tumour cell - stroma interface. The interaction between tumour cells and stromal fibroblasts mentioned above [38] might play an important role in this counterregulatory mechanism under HA deprived conditions as it was shown for breast carcinomas that the tumour-adjacent stroma showed elevated levels of HA and hyaluronectin to facilitate invasion [39]. However, despite the utilisation of stromal HA the current findings clearly showed that tumour cell mediated HA synthesis is critical in this model of ESCC.

In contrast RHAMM remained more evenly distributed after both interventions. The previous characterisation of the molecular mechanisms underlying the inhibition of malignant ESCC phenotype by interference with HA synthesis *in vitro *suggested that both RHAMM and CD44 signalling are critically involved in the proliferative and migratory phenotype of ESCC [24] through activation of focal adhesion signalling and MAPK signalling. The abundant expression of RHAMM and the redistribution of CD44 upon treatment in xenograft tumours are therefore in line with the proposed role of RHAMM and CD44 in transducing the effects of HA in this model. In addition, in prostate carcinoma HAS3 and HAS2 have been shown to produce HA that is broken down by Hyal1 and that subsequently drives tumour progression and even metastasis [5,40,41]. Therefore, degradation of the high molecular weight HA into smaller fragments may contribute to tumour progression in ESCC and should be investigated in future studies.

Remarkably, the EGF receptor (EGFR, ErbB1) is overexpressed in 40% to 90% of ESCC tumours and overexpression of EGFR is associated with a poor prognosis [42,43]. As we show here, EGFR expression is positively correlated with HAS3 expression in human ESCC. Of note, a steeper correlation between HAS3 and EGFR levels was found in the subgroup of T = 1 tumours, which possibly suggests a stronger dependence of this early tumour stage on EGF stimulated HAS3 expression. In line with this finding, EGF receptor activation led to induction of HAS3 in ESCC. Induction of HAS3 expression by EGF and ErbB2 receptors has also been shown for keratinocytes, prostate and lung carcinoma cells [44-47]. Therefore, EGF may be an important regulator of HAS3 expression in ESCC, which would be especially relevant in cancers known to be responsive to EGF inhibition, such as head and neck squamous cell carcinoma and metastatic colorectal cancer.

On the other hand, HA has been shown to contribute to the EGFR pathway via HA-CD44 interaction. HA-CD44 complexes colocalize and potentially transactivate the EGF receptor leading to phosphorylation of ERK1 and ERK2 in glioblastoma cell lines [48] and to increase tumour growth, migration and resistance to a variety of chemotherapeutic drugs such as methotrexate, doxorubicin, adriamycin and cisplatin in head and neck cancer [49]. In line with this, reduction of HA synthesis by 4-MU enhances the anticancer activity of gemcitabine in pancreatic cancer cells [12]. Consistently, adding exogenous HA leads to increased resistance to the EGFR inhibitor gefitinib in non small lung cancer cells [47]. However, vice versa, EGFR was also shown to modify the HA induced expression of a number of genes associated with cellular invasion and proliferation i.e. plasminogen activator inhibitor-1 (PAI-1) or tissue inhibitor of metalloproteinases (TIMP-1) in glioblastoma cell lines [48]. Moreover, in corneal epithelial cells, it was shown that HA and EGFR effects on migration were additive and that inhibition of either HA or EGFR signalling could not completely abolish the combined effects. This observation might indicate additional independent actions of EGFR and HA-CD44 [50]. Taken together, these reports show a close interrelationship between EGFR and HA-CD44 pathways and possibly a positive regulatory feedback in which EGF induces HA production which in turn amplifies the EGFR dependent signalling via CD44. Therefore, therapeutic modulation of the HA system may contribute new anticancer strategies in tumours dependent on EGFR signalling by disruption of this feedback cycle.

## Conclusions

In summary the present data extend the results from cell culture experiments [24] to *in vivo *growth of human oesophageal xenograft tumours. Specifically, it is proposed that ESCC tumour cells overexpress HAS3 in an EGFR dependent manner and that this overexpression supports a dedifferentiated proliferative tumour cell phenotype. Therefore, pharmacologic inhibition of HA synthesis may provide a novel therapeutic target for ESCC.

## Competing interests

The authors declare that they have no competing interests.

## Authors' contributions

ST planned and carried out the cell culture and lentiviral experiments, supervised the animal studies and wrote the manuscript. TF carried out and supervised the animal experiments. EP performed the histological staining and analysed them. GD designed primers and performed real time PCR experiments. KJ and CD carried out the animal vCT experiments. FA analysed the vCT experiments and did counselling. KP, WTK, NHS acquired the human oesophageal cancer samples, performed staging and did counselling. RCS provided the RHAMM antibody and participated in the planning of the experiments. BH participated in the design of the study and did counselling regarding growth factors. JWF designed and coordinated the study and drafted the manuscript. All authors read and approved the final manuscript.

## References

[B1] Parkin DM Bray F Ferlay J Pisani P Global cancer statistics, 2002 CA Cancer J Clin 2005 55 74 7108 10.3322/canjclin.55.2.74 15761078

[B2] Enzinger PC Mayer RJ Esophageal cancer N Engl J Med 2003 349 2241 2252 10.1056/NEJMra035010 14657432

[B3] Cooper JS Guo MD Herskovic A Macdonald JS Martenson JA Al-Sarraf M Byhardt R Russell AH Beitler JJ Spencer S Chemoradiotherapy of locally advanced esophageal cancer: long-term follow-up of a prospective randomized trial (RTOG 85-01). Radiation Therapy Oncology Group JAMA 1999 281 1623 1627 10.1001/jama.281.17.1623 10235156

[B4] Itano N Sawai T Yoshida M Lenas P Yamada Y Imagawa M Shinomura T Hamaguchi M Yoshida Y Ohnuki Y Three isoforms of mammalian hyaluronan synthases have distinct enzymatic properties J Biol Chem 1999 274 25085 25092 10.1074/jbc.274.35.25085 10455188

[B5] Bharadwaj AG Rector K Simpson MA Inducible hyaluronan production reveals differential effects on prostate tumor cell growth and tumor angiogenesis J Biol Chem 2007 282 20561 20572 10.1074/jbc.M702964200 17502371

[B6] Li Y Li L Brown TJ Heldin P Silencing of hyaluronan synthase 2 suppresses the malignant phenotype of invasive breast cancer cells Int J Cancer 2007 120 2557 2567 10.1002/ijc.22550 17315194

[B7] Udabage L Brownlee GR Nilsson SK Brown TJ The over-expression of HAS2, Hyal-2 and CD44 is implicated in the invasiveness of breast cancer Exp Cell Res 2005 310 205 217 10.1016/j.yexcr.2005.07.026 16125700

[B8] Tofuku K Yokouchi M Murayama T Minami S Komiya S HAS3-related hyaluronan enhances biological activities necessary for metastasis of osteosarcoma cells Int J Oncol 2006 29 175 183 16773198

[B9] Kim HR Wheeler MA Wilson CM Iida J Eng D Simpson MA McCarthy JB Bullard KM Hyaluronan facilitates invasion of colon carcinoma cells in vitro via interaction with CD44 Cancer Res 2004 64 4569 4576 10.1158/0008-5472.CAN-04-0202 15231668

[B10] Kakizaki I Kojima K Takagaki K Endo M Kannagi R Ito M Maruo Y Sato H Yasuda T Mita S A novel mechanism for the inhibition of hyaluronan biosynthesis by 4-methylumbelliferone J Biol Chem 2004 279 33281 33289 10.1074/jbc.M405918200 15190064

[B11] Yoshihara S Kon A Kudo D Nakazawa H Kakizaki I Sasaki M Endo M Takagaki K A hyaluronan synthase suppressor, 4-methylumbelliferone, inhibits liver metastasis of melanoma cells FEBS Lett 2005 579 2722 2726 10.1016/j.febslet.2005.03.079 15862315

[B12] Nakazawa H Yoshihara S Kudo D Morohashi H Kakizaki I Kon A Takagaki K Sasaki M 4-methylumbelliferone, a hyaluronan synthase suppressor, enhances the anticancer activity of gemcitabine in human pancreatic cancer cells Cancer Chemother Pharmacol 2006 57 165 170 10.1007/s00280-005-0016-5 16341905

[B13] Palyi-Krekk Z Barok M Isola J Tammi M Szollosi J Nagy P Hyaluronan-induced masking of ErbB2 and CD44-enhanced trastuzumab internalisation in trastuzumab resistant breast cancer Eur J Cancer 2007 43 2423 2433 10.1016/j.ejca.2007.08.018 17911008

[B14] Lokeshwar VB Lopez LE Munoz D Chi A Shirodkar SP Lokeshwar SD Escudero DO Dhir N Altman N Antitumor activity of hyaluronic acid synthesis inhibitor 4-methylumbelliferone in prostate cancer cells Cancer Res 2010 70 2613 2623 10.1158/0008-5472.CAN-09-3185 20332231 PMC2848908

[B15] Toole BP Hyaluronan: from extracellular glue to pericellular cue Nat Rev Cancer 2004 4 528 539 10.1038/nrc1391 15229478

[B16] Liu N Gao F Han Z Xu X Underhill CB Zhang L Hyaluronan synthase 3 overexpression promotes the growth of TSU prostate cancer cells Cancer Res 2001 61 5207 5214 11431361

[B17] Kim S Takahashi H Lin W-W Descargues P Grivennikov S Kim Y Luo J-L Karin M Carcinoma-produced factors activate myeloid cells through TLR2 to stimulate metastasis Nature 2009 457 102 106 10.1038/nature07623 19122641 PMC2746432

[B18] Itano N Zhuo L Kimata K Impact of the hyaluronan-rich tumor microenvironment on cancer initiation and progression Cancer Sci 2008 99 1720 1725 10.1111/j.1349-7006.2008.00885.x 18564137 PMC11159524

[B19] Koyama H Kobayashi N Harada M Takeoka M Kawai Y Sano K Fujimori M Amano J Ohhashi T Kannagi R Significance of tumor-associated stroma in promotion of intratumoral lymphangiogenesis: pivotal role of a hyaluronan-rich tumor microenvironment Am J Pathol 2008 172 179 193 10.2353/ajpath.2008.070360 18079437 PMC2189609

[B20] Ropponen K Tammi M Parkkinen J Eskelinen M Tammi R Lipponen P Agren U Alhava E Kosma VM Tumor cell-associated hyaluronan as an unfavorable prognostic factor in colorectal cancer Cancer Res 1998 58 342 347 9443415

[B21] Lipponen P Aaltomaa S Tammi R Tammi M Agren U Kosma VM High stromal hyaluronan level is associated with poor differentiation and metastasis in prostate cancer Eur J Cancer 2001 37 849 856 10.1016/S0959-8049(00)00448-2 11313172

[B22] Wang C Tammi M Guo H Tammi R Hyaluronan distribution in the normal epithelium of esophagus, stomach, and colon and their cancers Am J Pathol 1996 148 1861 1869 8669472 PMC1861659

[B23] Camenisch TD Spicer AP Brehm-Gibson T Biesterfeldt J Augustine ML Calabro A Kubalak S Klewer SE McDonald JA Disruption of hyaluronan synthase-2 abrogates normal cardiac morphogenesis and hyaluronan-mediated transformation of epithelium to mesenchyme J Clin Invest 2000 106 349 360 10.1172/JCI10272 10930438 PMC314332

[B24] Twarock S Tammi MI Savani RC Fischer JW Hyaluronan stabilizes focal adhesions, filopodia, and the proliferative phenotype in esophageal squamous carcinoma cells J Biol Chem 2010 285 23276 23284 10.1074/jbc.M109.093146 20463012 PMC2906320

[B25] Sarbia M Bosing N Hildebrandt B Koldovsky P Gerharz CD Gabbert HE Characterization of two newly established cell lines derived from squamous cell carcinomas of the oesophagus Anticancer Res 1997 17 2185 2192 9216685

[B26] Missbach-Guentner J Dullin C Kimmina S Zientkowska M Domeyer-Missbach M Malz C Grabbe E Stuhmer W Alves F Morphologic changes of mammary carcinomas in mice over time as monitored by flat-panel detector volume computed tomography Neoplasia 2008 10 663 673 18592006 10.1593/neo.08270 PMC2435003

[B27] Pavan L Tarrade A Hermouet A Delouis C Titeux M Vidaud M Therond P Evain-Brion D Fournier T Human invasive trophoblasts transformed with simian virus 40 provide a new tool to study the role of PPARgamma in cell invasion process Carcinogenesis 2003 24 1325 1336 10.1093/carcin/bgg074 12807721

[B28] Twarock S Rock K Sarbia M Weber AA Janicke RU Fischer JW Synthesis of hyaluronan in oesophageal cancer cells is uncoupled from the prostaglandin-cAMP pathway Br J Pharmacol 2009 157 234 243 10.1111/j.1476-5381.2009.00138.x 19338584 PMC2697807

[B29] Zhang B Xia HQ Cleghorn G Gobe G West M Wei MQ A highly efficient and consistent method for harvesting large volumes of high-titre lentiviral vectors Gene Ther 2001 8 1745 1751 10.1038/sj.gt.3301587 11892843

[B30] Itano N Atsumi F Sawai T Yamada Y Miyaishi O Senga T Hamaguchi M Kimata K Abnormal accumulation of hyaluronan matrix diminishes contact inhibition of cell growth and promotes cell migration Proc Natl Acad Sci USA 2002 99 3609 3614 10.1073/pnas.052026799 11891291 PMC122571

[B31] Tammi RH Kultti A Kosma V-M Pirinen R Auvinen P Tammi MI Hyaluronan in human tumors: pathobiological and prognostic messages from cell-associated and stromal hyaluronan Semin Cancer Biol 2008 18 288 295 10.1016/j.semcancer.2008.03.005 18468453

[B32] Kosunen A Ropponen K Kellokoski J Pukkila M Virtaniemi J Valtonen H Kumpulainen E Johansson R Tammi R Tammi M Reduced expression of hyaluronan is a strong indicator of poor survival in oral squamous cell carcinoma Oral Oncol 2004 40 257 263 10.1016/j.oraloncology.2003.08.004 14747056

[B33] Pirinen R Tammi R Tammi M Hirvikoski P Parkkinen JJ Johansson R Bohm J Hollmen S Kosma VM Prognostic value of hyaluronan expression in non-small-cell lung cancer: Increased stromal expression indicates unfavorable outcome in patients with adenocarcinoma Int J Cancer 2001 95 12 17 10.1002/1097-0215(20010120)95:1<12::AID-IJC1002>3.0.CO;2-E 11241304

[B34] Setala LP Tammi MI Tammi RH Eskelinen MJ Lipponen PK Agren UM Parkkinen J Alhava EM Kosma VM Hyaluronan expression in gastric cancer cells is associated with local and nodal spread and reduced survival rate Br J Cancer 1999 79 1133 1138 10.1038/sj.bjc.6690180 10098747 PMC2362238

[B35] Xing R Regezi JA Stern M Shuster S Stern R Hyaluronan and CD44 expression in minor salivary gland tumors Oral Dis 1998 4 241 247 10.1111/j.1601-0825.1998.tb00287.x 10200702

[B36] Bohm J Niskanen L Tammi R Tammi M Eskelinen M Pirinen R Hollmen S Alhava E Kosma VM Hyaluronan expression in differentiated thyroid carcinoma J Pathol 2002 196 180 185 10.1002/path.1032 11793369

[B37] Edward M Gillan C Micha D Tammi RH Tumour regulation of fibroblast hyaluronan expression: a mechanism to facilitate tumour growth and invasion Carcinogenesis 2005 26 1215 1223 10.1093/carcin/bgi064 15746159

[B38] Knudson W Biswas C Li XQ Nemec RE Toole BP The role and regulation of tumour-associated hyaluronan Ciba Found Symp 1989 143 150 159 discussion 159-169, 281-155 2680343 10.1002/9780470513774.ch10

[B39] Bertrand P Girard N Delpech B Duval C d'Anjou J Dauce JP Hyaluronan (hyaluronic acid) and hyaluronectin in the extracellular matrix of human breast carcinomas: comparison between invasive and non-invasive areas Int J Cancer 1992 52 1 6 10.1002/ijc.2910520102 1379993

[B40] Bharadwaj AG Kovar JL Loughman E Elowsky C Oakley GG Simpson MA Spontaneous metastasis of prostate cancer is promoted by excess hyaluronan synthesis and processing Am J Pathol 2009 174 1027 1036 10.2353/ajpath.2009.080501 19218337 PMC2665762

[B41] Simpson MA Concurrent expression of hyaluronan biosynthetic and processing enzymes promotes growth and vascularization of prostate tumors in mice Am J Pathol 2006 169 247 257 10.2353/ajpath.2006.060032 16816377 PMC1698770

[B42] Ozawa S Ueda M Ando N Abe O Shimizu N High incidence of EGF receptor hyperproduction in esophageal squamous-cell carcinomas Int J Cancer 1987 39 333 337 10.1002/ijc.2910390311 3493224

[B43] Shimada Y Imamura M Watanabe G Uchida S Harada H Makino T Kano M Prognostic factors of oesophageal squamous cell carcinoma from the perspective of molecular biology Br J Cancer 1999 80 1281 1288 10.1038/sj.bjc.6690499 10376985 PMC2362359

[B44] Bourguignon LY Gilad E Peyrollier K Heregulin-mediated ErbB2-ERK signaling activates hyaluronan synthases leading to CD44-dependent ovarian tumor cell growth and migration J Biol Chem 2007 282 19426 19441 10.1074/jbc.M610054200 17493932

[B45] Pasonen-Seppanen S Karvinen S Torronen K Hyttinen JM Jokela T Lammi MJ Tammi MI Tammi R EGF upregulates, whereas TGF-beta downregulates, the hyaluronan synthases Has2 and Has3 in organotypic keratinocyte cultures: correlations with epidermal proliferation and differentiation J Invest Dermatol 2003 120 1038 1044 10.1046/j.1523-1747.2003.12249.x 12787132

[B46] Pasonen-Seppanen SM Maytin EV Torronen KJ Hyttinen JM Hascall VC MacCallum DK Kultti AH Jokela TA Tammi MI Tammi RH All-trans retinoic acid-induced hyaluronan production and hyperplasia are partly mediated by EGFR signaling in epidermal keratinocytes J Invest Dermatol 2008 128 797 807 10.1038/sj.jid.5701098 17943186

[B47] Chow G Tauler J Mulshine JL Cytokines and growth factors stimulate hyaluronan production: role of hyaluronan in epithelial to mesenchymal-like transition in non-small cell lung cancer J Biomed Biotechnol 2010 2010 485468 20671927 10.1155/2010/485468 PMC2910509

[B48] Tsatas D Kanagasundaram V Kaye A Novak U EGF receptor modifies cellular responses to hyaluronan in glioblastoma cell lines J Clin Neurosci 2002 9 282 288 10.1054/jocn.2001.1063 12093135

[B49] Wang SJ Bourguignon LY Hyaluronan and the interaction between CD44 and epidermal growth factor receptor in oncogenic signaling and chemotherapy resistance in head and neck cancer Arch Otolaryngol Head Neck Surg 2006 132 771 778 10.1001/archotol.132.7.771 16847188

[B50] Nishida T Nakamura M Mishima H Otori T Hyaluronan stimulates corneal epithelial migration Exp Eye Res 1991 53 753 758 10.1016/0014-4835(91)90110-Z 1783012

